# Supplementary material for: All-optical structuring of laser-driven proton beam profiles
Source: Nat Commun. 2018 Dec 13;9:5292. doi: 10.1038/s41467-018-07756-z (PMC6294339; doi:10.1038/s41467-018-07756-z)
Supplement: Supplementary file 1 — Supplementary Information [file 41467_2018_7756_MOESM1_ESM.pdf]

Supplementary Material to Article: "All-optical structuring of laser-driven proton beam profiles"

Lieselotte Obst-Huebl,[1,2,*] Tim Ziegler,[1,2] Florian-Emanuel Brack,[1,2] João Branco,[1,2] Michael Bussmann,[1] Thomas E. Cowan,[1,2] Chandra B. Curry,[3,4] Frederico Fiuza,[3] Marco Garten,[1,2] Maxence Gauthier,[3] Sebastian Göde,[5] Siegfried H. Glenzer,[3] Axel Huebl,[1,2] Arie Irman,[1] Jongjin B. Kim,[3] Thomas Kluge,[1] Stephan D. Kraft,[1] Florian Kroll,[1] Josefine Metzkes-Ng,[1] Richard Pausch,[1,2] Irene Prencipe,[1] Martin Rehwald,[1,2] Christian Roedel,[6] Hans-Peter Schlenvoigt,[1] Ulrich Schramm,[1,2] and Karl Zeil[1]

[1]*Helmholtz-Zentrum Dresden - Rossendorf, Institute of Radiation Physics, Bautzner Landstr. 400, 01328 Dresden, Germany*
[2]*Technische Universität Dresden, 01062 Dresden, Germany*
[3]*High Energy Density Science Division, SLAC National Accelerator Laboratory, Menlo Park, California 94025, USA*
[4]*University of Alberta, Edmonton, Alberta T6G 1H9, Canada*
[5]*European XFEL GmbH, Holzkoppel 4, 22869 Schenefeld, Germany*
[6]*Helmholtz Institute Jena, Fröbelstieg 3, 07743 Jena, Germany*

SUPPLEMENTARY NOTE 1

In this Supplementary Note spatial resolution limits of imprinted features are discussed. Provided the residual gas density and, consequently, the laser induced fields are high enough to allow for effective imprinting of laser intensity features in the proton beam profile, limitations exist to the minimum size of these features. Distinct electro-static field structures that largely correspond to the laser intensity profile are only induced in the case that the lateral electron density gradient at the border of a low density plasma column is sufficiently short compared to the overall diameter of the column, approximately represented by the fixed ion population (ref. to insets in Suppl. Fig. 1). The transverse extent of the latter results from the size of the ionizing intensity feature. If, however, electrons are distributed over a significantly larger area, the electro-static field pattern and the corresponding proton beam deflections are blurred. The lateral extent of the electron density gradient is dominated by the average electron kinetic energy, often called temperature, which depends on the laser intensity expressed via the dimensionless vector potential a_0. Here, the plasma Debye length $\lambda_\mathrm{D}(z)$ is used as a characteristic length of the electron density gradient to be compared with the size of intensity features as a function of the distance z from the target plane (depicted in Suppl. Fig. 1). Note that, since simulations performed for this work indicate a systematically longer density gradient than the Debye length for the given laser and density parameters (refer to Fig. 3 of the main article), this comparison can only serve to explore general trends. As displayed in Suppl. Fig. 1, the laser beam diameter decreases when approaching the laser focus according to the beam divergence, the laser intensity increases and so does $\lambda_\mathrm{D} \sim \sqrt{a_0}$. Once an intensity feature of diameter $d(z)$ along z becomes smaller than $2 \times \lambda_\mathrm{D}(z)$ (accounting for electron density gradients on both sides of the 1-dimensional ion population), electro-static fields at the border between electron and ion population become less distinct. As electrons are increasingly expelled by the laser, space-charge fields would then mainly arise from the remaining ion population, which is expected to result in a different, less pronounced signature in the deflection of TNSA protons. This spatial resolution criterion for a clear projection of laser intensity features to sharp electric fields at the border of the ion population poses a lower limit for the field length L over which TNSA protons are subject to deflection during laser profile imprinting. As will need to be confirmed in experiments, features that remain smaller than $2 \times \lambda_\mathrm{D}(z)$ over the entire ionized region (with a length of 14 - 15 mm for our laser conditions) therefore should not reappear in the proton beam profile. Longer electron density gradients are equivalent to an increase in the spatial resolution limit, meaning that only larger spatial intensity features can be imprinted in the proton beam profile.

SUPPLEMENTARY NOTE 2

This Supplementary Note discusses the course of the electric field strength along the proton track in region II. It was first estimated assuming an exponential electron density gradient and using an analytical description to calculate front fields at the plasma-vacuum interface.[1] The results are displayed in Suppl. Fig. 2a) (solid lines) for the two

* l.obst-huebl@hzdr.de

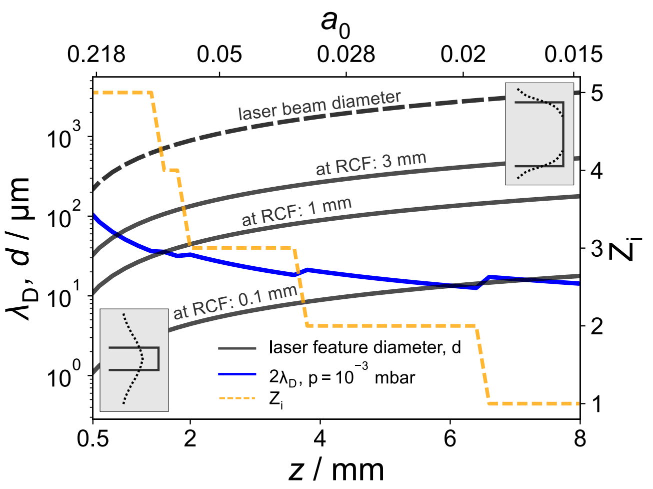

Supplementary Fig. 1. Spatial resolution criterion of imprinted features. Displayed is the scaling of the lateral extent of the electron population to both sides of the low density plasma column represented by $2 \times \lambda_{\mathrm{D}}$ (blue line) versus exemplary feature diameters d (gray lines, annotated are the feature diameters at the position of radio-chromic film stacks positioned 45 mm downstream of the laser focus) scaled geometrically along the propagation length z and the corresponding laser vector potential a_0. For reference, ionization states Z_{i} of N (dashed orange line) are given. Insets schematically depict electron and ion distributions leading to distinct (upper right) and blurred (lower left) electro-static field structures. Feature diameters larger than $2 \times \lambda_{\mathrm{D}}$ (blue line) are resolvable and can be imprinted in the proton beam profile.

residual gas density cases realized in the tungsten wire experiment. For a constant residual gas density, the field strength changes only roughly one order of magnitude over the course of ~ 8 mm for $z \geq 500$ µm. Changing the gas density by two orders of magnitude in the investigated density regime results in roughly a factor 10 in field strength. Steps in the course of the electric field strength correspond to a change in ionization state (ref. to Suppl. Fig. 1 for comparison) due to barrier suppression ionization[2] by the transmitted laser beam, the intensity of which decreases according to the laser opening angle. Collisional ionization plays no role in this very low density regime, as electron-ion collisions only occur at a maximum rate of 0.1 to 10 ms^{-1} at the given electron temperatures of 10 to few 100 eV.[3] Simulations varying a_0 and a sinusoidal laser intensity modulation corresponding to the feature diameter d were conducted. As the analytically deduced electric field strength was found to scale only weakly with ionization state along z, simulations were performed with Hydrogen at a density corresponding to three to four-fold ionization of Nitrogen at the two vacuum chamber pressure cases realized in the tungsten wire experiment. Calculated values of the electric field strength at the selected Hydrogen gas densities (dashed lines in Suppl. Fig. 2a) showed a sufficiently similar course as those of the experimental residual gas settings (solid lines in Suppl. Fig. 2a). The resulting maximum field strengths E_{trans}, oriented transversally to the proton propagation direction, are displayed in Suppl. Fig. 2b). Apart from the region close to the target at $z < 500$ µm, values of E_{trans} are largely constant over a distance of several mm along z. Absolute field strength values differ from the analytical results, which is attributed to a significantly longer electron density gradient found in simulations than the calculated Debye length, as mentioned in the previous paragraph. The simulations and analytical calculations confirm the conception of fairly modest changes in $E_{\mathrm{trans}}(z)$ along region II of the proton deflection scheme introduced in the main manuscript.

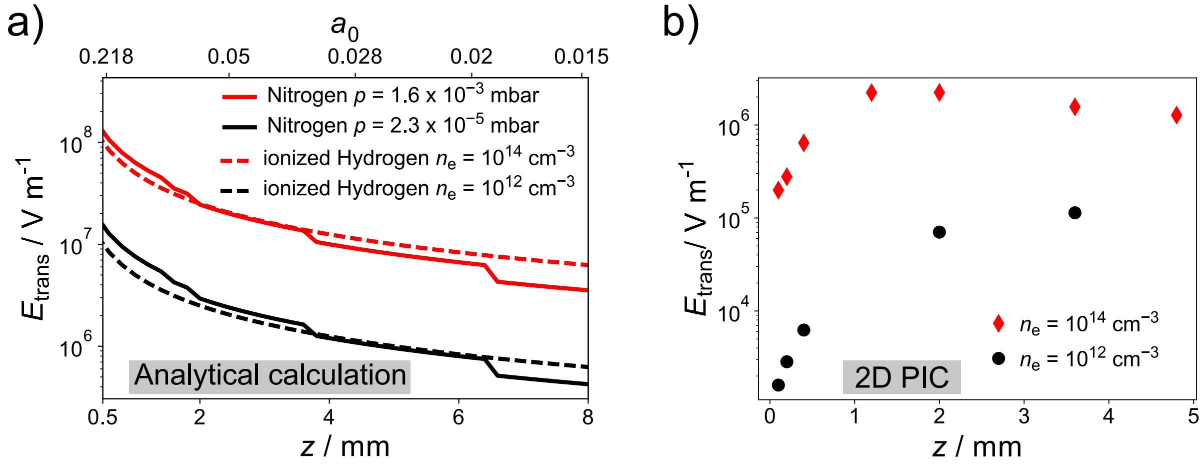

Supplementary Fig. 2. Course of the electric field strength along region II. a) Analytical calculation of the maximum field strength at the border of a low density plasma column assuming an exponential electron density gradient.[1] Solid lines correspond to residual gas pressures p realized in the tungsten wire experiments. For simplicity the residual gas is assumed to consist only of Nitrogen. Dashed lines correspond to the respective Hydrogen densities used for PIC simulations. Both Nitrogen and Hydrogen values agree reasonably well for a given density setting. b) Maximum field strengths E_{trans} from PIC simulations, varying laser conditions along z for two different residual gas electron densities. Every data point represents one simulation at 21 ps after initialization of the laser pulse in the respective window.

SUPPLEMENTARY REFERENCES

[1] Mora, P. Plasma Expansion into a Vacuum. *Phys. Rev. Lett.*, **90**, 185002 (2003). URL `https://journals.aps.org/prl/abstract/10.1103/PhysRevLett.90.185002`.

[2] Mulser, P. & Bauer, D. *High power laser-matter interaction* (Springer-Verlag Berlin Heidelberg, 2010).

[3] Dendy, R. *Plasma physics: an introductory course* (Cambridge University Press, 1993).
